# Low Vitamin D Levels and Frailty Status in Older Adults: A Systematic Review and Meta-Analysis

**DOI:** 10.3390/nu12082286

**Published:** 2020-07-30

**Authors:** Diego Marcos-Pérez, María Sánchez-Flores, Stefania Proietti, Stefano Bonassi, Solange Costa, Joao Paulo Teixeira, Juan Fernández-Tajes, Eduardo Pásaro, Vanessa Valdiglesias, Blanca Laffon

**Affiliations:** 1 Grupo DICOMOSA, Centro de Investigaciones Científicas Avanzadas (CICA), Departamento de Psicología, Facultad de Ciencias de la Educación, Campus Elviña s/n, Universidade da Coruña, 15071 A Coruña, Spain; diego.marcos@udc.es (D.M.-P.); maria.sanchez@udc.es (M.S.-F.); pspasaro@udc.es (E.P.); blaffon@udc.es (B.L.); 2 Instituto de Investigación Biomédica de A Coruña (INIBIC), AE CICA-INIBIC, Oza, 15071 A Coruña, Spain; 3 Environmental Health Department, National Health Institute, Rua Alexandre Herculano 321, 4000-055 Porto, Portugal; solange.costa2@gmail.com (S.C.); jpft12@gmail.com (J.P.T.); 4 EPIUnit—Instituto de Saúde Pública, Universidade do Porto, Rua das Taipas, no 135, 4050-600 Porto, Portugal; 5 Scientific Direction, IRCCS San Raffaele Pisana, Via di Val Cannuta, 247, 00166 Rome, Italy; stefania.proietti@sanraffaele.it; 6 Unit of Clinical and Molecular Epidemiology, IRCCS San Raffaele Pisana, Via di Val Cannuta, 247, 00166 Rome, Italy; stefano.bonassi@sanraffaele.it; 7 Department of Human Sciences and Quality of Life Promotion, San Raffaele University, Via di Val Cannuta, 247, 00166 Rome, Italy; 8 Wellcome Centre for Human Genetics, McCarthy’s group, University of Oxford, Roosevelt Drive, Headington, Oxford OX3 7BN, UK; jfertaj@well.ox.ac.uk; 9 Genetic and Molecular Epidemiology Unit, Department of Clinical Sciences, CRC, SUS Malmö, Jan Waldenströms gata 35, House 91:12, SE-214 28 Malmö, Sweden; 10 Grupo DICOMOSA, Centro de Investigaciones Científicas Avanzadas (CICA), Departamento de Biología, Facultad de Ciencias, Campus A Zapateira s/n, Universidade da Coruña, 15071 A Coruña, Spain

**Keywords:** frailty, meta-analysis, older adults, systematic review, vitamin D

## Abstract

Serum vitamin D deficiency is widespread among older adults and is a potential modifiable risk factor for frailty. Moreover, frailty has been suggested as an intermediate step in the association between low levels of vitamin D and mortality. Hence, we conducted a systematic review of the literature and meta-analysis to test the possible association of low concentrations of serum 25-hydroxyvitamin D (25(OH)D), a marker of vitamin D status, with frailty in later life. We reviewed cross-sectional or longitudinal studies evaluating populations of older adults and identifying frailty by a currently validated scale. Meta-analyses were restricted to cross-sectional data from studies using Fried’s phenotype to identify frailty. Twenty-six studies were considered in the qualitative synthesis, and thirteen studies were included in the meta-analyses. Quantitative analyses showed significant differences in the comparisons of frail (standardized mean difference (SMD)—1.31, 95% confidence interval (CI) (−2.47, −0.15), *p* = 0.0271) and pre-frail (SMD—0.79, 95% CI (−1.58, −0.003), *p* = 0.0491) subjects vs. non-frail subjects. Sensitivity analyses reduced heterogeneity, resulting in a smaller but still highly significant between-groups difference. Results obtained indicate that lower 25(OH)D levels are significantly associated with increasing frailty severity. Future challenges include interventional studies testing the possible benefits of vitamin D supplementation in older adults to prevent/palliate frailty and its associated outcomes.

## 1. Introduction

Vitamin D is a fat soluble secosteroid hormone which is mainly produced endogenously in the skin (80–90%) after sunshine exposure. The other sources of vitamin D (10–20%) are the intake of food which naturally contains this vitamin and dietary supplements [1]. Vitamin D has a plethora of functions; the major physiologic function is regulating calcium and phosphate homeostasis as well as mineral bone metabolism. However, vitamin D also plays an extensive role as a cell differentiating and antiproliferative factor with actions in a variety of tissues, including the renal, cardiovascular, and immune systems [2,3].

Although biologically inert, 25-hydroxyvitamin D (25(OH)D) is the major circulating metabolite of vitamin D and is globally accepted as a marker of vitamin D status. There is no consensus about the cut-offs of this metabolite to describe an individual’s vitamin D status. Thus, the Institute of Medicine and the Endocrine Society have different definitions for vitamin D deficiency and optimal values [4,5]. The prevalence of serum 25(OH)D levels lower than 20 ng/mL in the US population was reported as 32% [6], and concentrations lower than 30 ng/mL were observed in 57.5% of men and in 60.7% of women in a Canadian cohort [7]. Variations in prevalence may be related to characteristics of the analyzed populations such as sex, age, skin pigmentation, or vitamin D supplementation, or even to the sampling season [6,7].

The prevalence of vitamin D deficiency is higher in older adults than in younger adults, and especially higher in institutionalized (30–90%) than in community-dwelling older people (2–60%) [8,9]. There are multiple causes of this higher prevalence in later life, including lower sunshine exposure owing to a reduction in outdoor activity, lower dermal synthesis of pre-vitamin D, inadequate vitamin D intake, and decreased kidney function (calcitriol, the biologically active form of vitamin D, is produced in the kidney proximal tubule) [10].

The older population is particularly at risk for clinical complications associated with vitamin D deficiency. Several cross-sectional studies linked low vitamin D status with an increased risk of type 1 and type 2 diabetes mellitus, cardiovascular disease, certain cancers, cognitive decline, depression, autoimmunity, allergy, and even frailty [1]. Frailty is a geriatric syndrome with multiple causes and contributors, characterized by diminished strength, endurance, and reduced physiologic function, which increases an individual’s vulnerability for developing negative health outcomes, increased dependency and/or death [11]. A plethora of frailty measurements are currently in existence; among them, the two frailty identification tools most commonly accepted and used in clinical settings and in research studies are the phenotypic model proposed by Fried et al. [12] and the deficit accumulation model developed by Mitnitski, Mogilner, and Rockwood [13]. Prevalence of frailty is highly variable and dependent on a number of factors (e.g., frailty identification criteria, gender, age or socio-economic conditions); the heterogeneity of factors associated with frailty is reflected by the wide variation of prevalence estimates, ranging from 4% to 59.1% [14].

A potential modifiable risk factor for frailty seems to be serum vitamin D deficiency which, as already mentioned, is widespread among older adults. Moreover, frailty has been suggested as an intermediate step in the association between low levels of vitamin D and mortality [15]. A clear inverse association between vitamin D level and frailty in older adult populations has been reported in several studies [16,17,18], although others did not find such association [19,20].

The diversity of: (i) frailty model components, (ii) definitions of vitamin D deficiency, and (iii) laboratory methods used to measure 25(OH)D may in part explain the divergence of findings among studies examining the association of vitamin D status with frailty and mortality. For these reasons, and in order to confirm the hypothesis that vitamin D deficiency may be related to frailty syndrome, we conducted a systematic review of the literature and meta-analysis to test the possible association of low levels of 25(OH)D with frailty status in later life. Two previous meta-analyses have been published exploring this association [21,22]. These studies included a smaller number of studies (four cross-sectional plus six longitudinal studies, and seven longitudinal studies, respectively), did not consider the condition of pre-frailty, and did not take into consideration a major source of variability such as the frailty identification criteria used in the original studies. For these reasons, together with the inclusion of additional recent studies analyzing vitamin D in frail subjects, a newly updated, more comprehensive and more homogeneous meta-analysis is required, which may provide quantitative indication about the level of 25(OH)D in frailty patients and in early stages of this syndrome.

## 2. Materials and Methods

The Preferred Reporting Items from Systematics Reviews and Meta-Analyses (PRISMA) statement [23] was followed to perform and report the present systematic review and meta-analyses.

### 2.1. Eligibility Criteria for the Systematic Review

Cross-sectional or longitudinal design observational studies conducted in humans, including populations of older adults (≥60 years old), and written in English or Spanish were considered eligible for this systematic review. In these studies, participant’s frailty had to be identified according to any of the currently validated scales for frailty identification. Review articles, editorials, commentaries, letters without data analysis, conference abstracts, studies conducted in animals, experimental studies, studies assessing populations with severe pathologies (cancer, diabetes, heart failure, etc.), considering frailty as a confounder, or employing a non-validated frailty identification tool were excluded.

### 2.2. Search Strategy

Studies included in this systematic review were identified through an extensive bibliographic search using the PubMed database (National Library of Medicine, National institutes of Health, Berthesda, MD, USA; http://www.ncbi.nih.gov/PubMed), updated to October 2018. The search was conducted by two independent researchers (D.M.-P. and V.V.) following a search strategy which comprised two terms intersected using the Boolean term “AND”. The search term included as the first descriptor was related to frailty (“frail*”), and the second one included descriptors related to vitamin D (“vitamin D” or “25(OH)D”). The search filter “Humans” was used to retrieve only studies conducted in human subjects. Initial screening was focused on title or abstract.

### 2.3. Data Collection Process

The following information was collected independently by two authors (D.M.-P. and V.V.) for each study complying with inclusion criteria: country of origin for the first author, type of study, size of the study population and the frailty groups, participant’s gender and age, criteria used to identify frailty, and outcomes (circulating concentrations of 25(OH)D) in each frailty group.

### 2.4. Studies Included in the Meta-Analysis

Studies included in the meta-analysis were those providing mean ± standard deviation (SD) data of 25(OH)D for frailty groups. Several studies provided quantitative parameters different than the mean, e.g., median and confidence intervals (CI) or inter-quartile range, or they classified the population according to 25(OH)D concentration ranges instead of by frailty status. In all these cases, emails were sent to corresponding authors asking for missing values, at least twice in a four-month period. Studies for which authors provided the requested data were also included in the meta-analyses. Meta-analyses were restricted to only those studies employing Fried’s criteria (original or modified version) to identify frailty. Only cross-sectional data were considered for the meta-analyses, since just six longitudinal studies which employed different frailty identification tools were available; hence, in longitudinal studies, just baseline values of 25(OH)D were included (follow-up values were dismissed).

### 2.5. Quality Assessment

Quality assessment of studies included in the meta-analyses was carried out by two independent investigators (D.M-.P. and V.V.), whilst a third reviewer was available for mediation (B.L.). The standard of study design was evaluated for each study by calculating a quality score (Q.S.) (Supplementary Material, Table S1). Each QS item scored from 1 to a maximum of 3 points based on reported data and matching status. The minimum and maximum of total QS possible was five and seventeen, respectively.

### 2.6. Statistical Analysis

All analyses were performed using the Comprehensive R Archive Network (https://cran.r-project.org/). Data were expressed as mean 25(OH)D ± SD. Heterogeneity across studies was evaluated using the I^2^ and tested with the Cochran Q chi-square statistics. Since all meta-analyses conducted showed statistically significant heterogeneity among studies (I^2^ ≥ 50% and *p* value < 0.05), the pooled standardized mean differences (SMD) with 95% confidence interval (95% CI) were estimated using a random-effects model with method according to Dersimonian and Laird [24]. Publication bias was assessed by visually inspecting funnel plots and using the Egger’s bias test. Whenever a significant result (*p* < 0.05) was found, the trim-and-fill method was used to adjust for any potential unpublished studies. The presence of confounders or effect modifiers was tested with a meta-regression analysis; the final model included year of publication and QS (as a continuous variable) as confounders. A sensitivity analysis was performed when a discrepant value of SMD in a single study was observed [25].

## 3. Results

### 3.1. Literature Search Results

**Figure 1:**
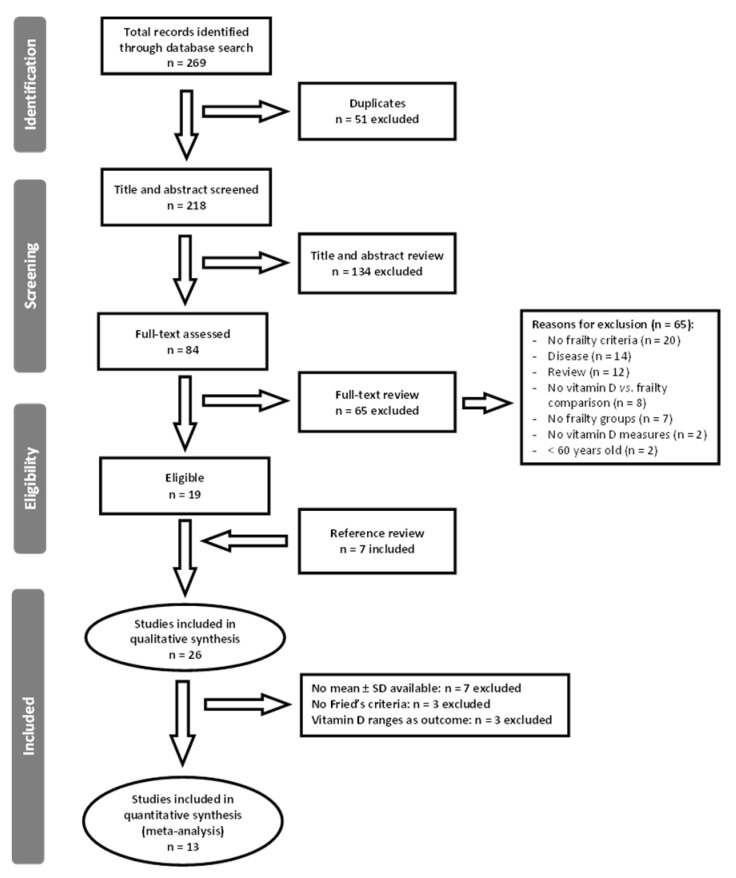
Preferred Reporting Items for Systematic Reviews and Meta-Analyses (PRISMA) flow chart of the study selection process. SD: standard deviation.

Two hundred and sixty-nine articles were initially identified in the literature search. After removal of duplicates, 218 articles were screened for potential eligibility (a flow chart is shown in Figure 1). Titles and abstracts were reviewed and 84 out of 218 were retrieved for full-text assessment of eligibility. Then, whole texts were completely reviewed, obtaining 19 studies which fitted the selection criteria. Seven additional publications were incorporated from the references section of published articles. Eventually, 26 studies fulfilled the inclusion criteria and were included in this review. After excluding studies not reporting mean + SD data or not employing Fried’s criteria for frailty identification, 13 studies were finally included in the meta-analysis.

### 3.2. Characteristics of Included Studies

Table 1 shows the characteristics of the 26 studies included in the systematic review (total sample size: 38,162 participants). None of the retrieved studies written in Spanish fulfilled the inclusion criteria. Eight out of 10 studies were performed with sample sizes above 300 participants, having a relatively high statistical power. Globally, percentages of individuals included in the three different frailty groups were 46.5% non-frail, 38% pre-frail and 15.5% frail participants. Prevalence of frailty and pre-frailty in the different studies ranged between 4% and 58%, and between 21% and 63%, respectively. Four studies reported both cross-sectional and longitudinal prospective evaluations (follow up periods from 2.9 to 12 years). Seven studies included females only, six studies included males only, and 13 included both genders. Europe and the USA were the main places where these studies were performed, with 46% and 35% of the total participants, respectively, followed by Asia and Australia (8% each), and only one study performed in Mexico. Focusing on European countries, four studies were conducted in Spain, two in Germany and the UK, and only one in the Netherlands, Portugal, Italy and France. According to the methodology employed to measure 25(OH)D, the radioreceptor assay, including radioimmunoassay, was used in half of the studies, while 15% of studies employed a chemiluminiscence assay. Frailty status was identified by using Fried’s criteria (original version or modified) in 80% of studies. Among them, 54% used the original version of frailty phenotype, and the rest of them introduced different modifications.

The minimum and maximum QS obtained in the studies included in the meta-analyses were seven and twelve, respectively (Table S1). Only one study included between 100 and 300 subjects; all other studies had more than 300 participants. Half of the studies were matched for age (difference between groups was less than five years), or for gender (studies not matched for this characteristic had a gender ratio either higher than 0.60 or lower than 0.40). In only two studies, frailty groups were balanced, and none of the studies described whether frailty was measured by geriatricians or specialized personnel. Approximately half of the studies (six studies) comprised only non-frail and frail groups, while the other half (seven studies) included also a pre-frail group.

### 3.3. Meta-Analysis Frailty Group vs. Non-Frailty Group

**Figure 2:**
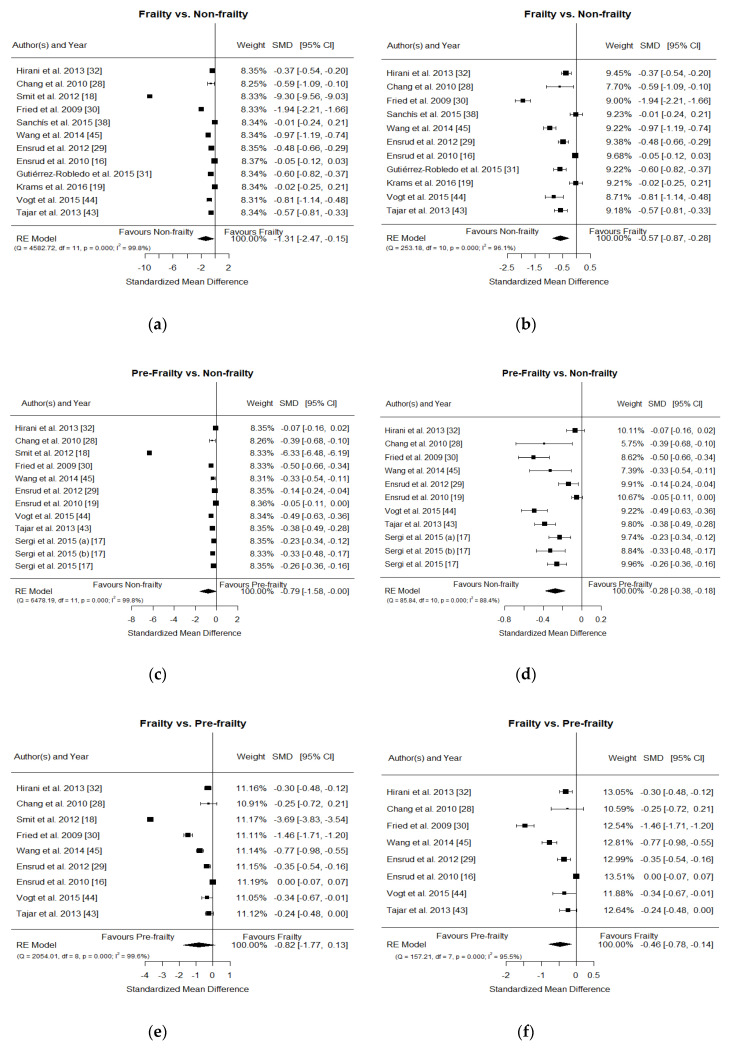
Forest plots for frailty vs. non-frailty comparison (**a**,**b**), pre-frailty vs. non-frailty comparison (**c**,**d**), and frailty vs. pre-frailty comparison (**e**,**f**); forest plots including all studies (**a**,**c**,**e**) and sensitivity analyses excluding Smit et al. [18] (**b**,**d**,**f**) are depicted. In order to respect original data provided by Sergi et al. [17], three populations from this study which differ in the number of positive criteria to classify pre-frail subjects were included in the meta-analyses comparing pre-frailty vs. non-frailty: pre-frail subjects with only one positive frailty criterion were embodied in “Sergi et al. 2015 (**a**) [17] ”; pre-frail subjects with two positive criteria were embodied in “Sergi et al. 2015 (**b**) [17]”; and all pre-frail subjects (with one and two positive criteria) were embodied in “Sergi et al. 2015 [17]”. RE: random-effects.

This meta-analysis included 12 studies out of the 13 suitable (one of the selected studies did not include a frail group) (see forest plot in Figure 2a). Heterogeneity was very high, even after removing most extreme results (96.05%, *p* < 0.0001) (Table 2; Figure 2b). No evidence of publication bias was found, and the concentration of 25(OH)D was significantly lower in the frailty group when compared to the non-frailty group.

### 3.4. Meta-Analysis Pre-Frailty Group vs. Non-Frailty Group

The ten studies included in this meta-analysis evaluated the association between levels of 25(OH)D and pre-frailty (Figure 2c). In agreement with the original data reported by Sergi et al. [17], all three criteria used to classify subjects as pre-frail, i.e., one, two, or one and two positive Fried’s frailty criteria, were considered in the meta-analysis. Heterogeneity was considerably reduced, but still significant (I^2^ = 88.4%, *p* < 0.0001) even after the extreme results by Smit et al. [18] were removed from the meta-analysis (Figure 2d). Also, in this case, a significantly lower level of 25(OH)D was observed in the group of pre-frail subjects when compared to non-frail subjects. The Egger’s regression test was not significant, indicating the absence of publication bias.

### 3.5. Meta-Analysis Frailty Group vs. Pre-Frailty Group

The meta-analysis comparing different degrees of frailty included nine studies (Figure 2e). Statistically significant heterogeneity among the studies was observed. Lower concentrations of 25(OH)D were observed in the frailty group, but this reduction reached statistical significance only when the study by Smit et al. [18] was removed from the comparison (Figure 2f). Egger’s test was not significant, suggesting no publication bias.

Meta-regression analyses did not show the presence of any significant effect modification or confounding in any of the comparisons conducted.

### 3.6. Sensitivity Analyses

**Figure 3:**
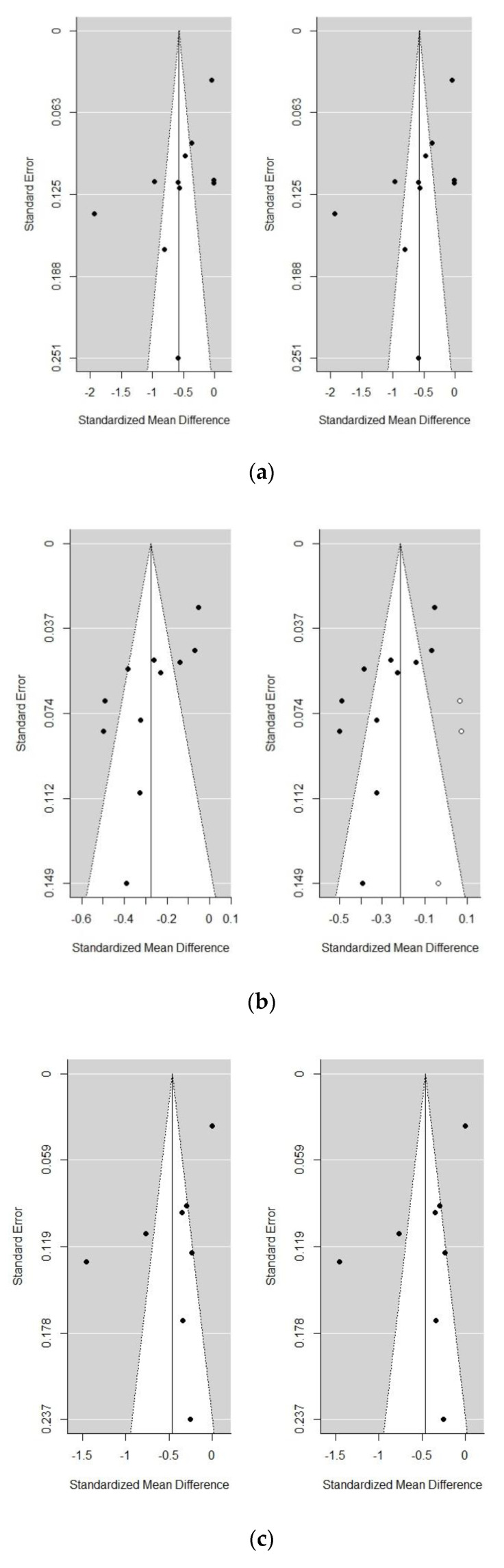
Funnel plots for 25(OH)D concentrations (sensitivity analyses excluding Smit et al. [18]). (**a**) frailty vs. non-frailty comparison, (**b**) pre-frailty vs. non-frailty comparison, and (**c**) frailty vs. pre-frailty comparison. Original data are shown on the left, and filled-in data in the trim-and-fill method on the right.

Considering the high heterogeneity among studies and the fact that the SMDs of the study conducted by Smit et al. [18] were notably discrepant with regard to all other studies included in the three comparisons reported in the forest plots (Figure 2a,c,e), with a much greater reduction of 25(OH)D in the study groups, we performed sensitivity analyses excluding this particular study in an attempt to reduce heterogeneity and to investigate how much of the overall effect was attributable to it. A summary of the results obtained with and without the study of Smit et al. [18] is reported in Table 2. In general, heterogeneity was reduced after removing this study, although it continued to be significant. Estimates of SMD obtained were lower than in the original analyses, but all of them reached statistical significance, indicating that 25(OH)D levels decrease while frailty severity increases. The presence of publication bias was only observed in the pre-frailty vs. non-frailty comparison; the estimate of the SMD was slightly changed but still significant after adjusting with the trim-and-fill method. Funnel plots for the three comparisons after excluding the study of Smit et al. [18] are shown in Figure 3.

## 4. Discussion

The current and unstoppable ageing of the world’s population leads to a constant increase of age-related disabilities, diseases, and frailty. These conditions are attracting the attention of researchers and governments who aim to reduce their effect on public health, mostly through prevention. As previously reported, vitamin D is an age-dependent biomarker associated with type 1 and type 2 diabetes mellitus [48,49,50], cardiovascular diseases [51,52], various types of cancers [1,53], neurological diseases [4,18], respiratory tract diseases [54], autoimmune diseases [55], and an increased risk of death [56], falls [57], and fractures [8].

Our literature search (26 studies) unequivocally showed an association of low 25(OH)D levels with frailty in almost all studies included in the review. Only one of them [19] failed to find a significant association between 25(OH)D concentration and frailty. The low proportion of non-frail subjects analyzed (6.12%), and the high proportions of both pre-frail (44.21%) and frail (49.7%) subjects, notably different to other studies with positive results, may have reduced the possibility of identifying an association between 25(OH)D and frailty.

Since there are a variety of tools to identify frailty, largely differing in their conceptualization of frailty, we decided to restrict the meta-analyses to those studies employing Fried’s criteria in order to increase homogeneity. Our results, which included 13 studies and a total of 20,355 study subjects, showed a clear and inverse relationship between 25(OH)D levels and frailty severity—as defined by the Fried’s phenotype—both in the original analyses and in the sensitivity analyses, which excluded the study by Smit et al. [18]. The exclusion of this study, motivated by results which showed a more pronounced difference between study groups, caused an obvious reduction of heterogeneity among the studies. Besides, the results obtained did not notably modify the original ones, but rather confirmed the association between vitamin D deficiency and frailty; the comparison between frail and pre-frail subjects resulted in a significant difference. The extreme results published by Smit et al. [18] can possibly be explained by the peculiarity of this research, i.e.,: (1) participants were recruited only from institutionalization settings; (2) Fried’s frailty criteria were largely modified (i.e., low body mass index instead of unintentional weight loss, muscular weakness determined by a question instead of grip strength measurement, and different questions to identify exhaustion and low physical activity); (3) a large proportion of participants were affected by chronic diseases: 66% of non-frail, 80% of pre-frail, and 94% of frail subjects, and the distribution of the presence of these chronic diseases was significantly different (*p* < 0.00001) among the three groups of individuals; and (4) the size of the frail group was not balanced with regard the other two groups (n = 453 frail individuals, n = 1915 pre-frail, n = 2363 non-frail individuals). All these conditions contribute to explain the stronger association found in this study and also justify the choice of removing the study in the sensitivity analyses.

Two previous systematic reviews and meta-analyses [21,22] were published on the relationship between vitamin D deficiency and frailty, with results consistent with the current ones, although these studies differ notably from the present one in several aspects of the design and analysis. The study from Zhou and colleagues [22] included only prospective cohort studies (n = 7, cross-sectional studies were excluded), and compared groups with the most extreme levels of 25(OH)D, calculating the pooled odds ratio of frailty in the lowest versus the highest level of 25(OH)D. Furthermore, in that meta-analysis, cut-off values for defining low and high level categories were different among studies, the number of categories were different (three or four depending on the study), and in some cases, the units used to measure 25(OH)D concentrations were also different. All these discrepancies may have led to a biased interpretation of the results, including an overestimation or an underestimation of the effect. Ju and colleagues [21] conducted a dose response meta-analysis, calculating the pooled risk estimate of frailty for a 25 nmol/L increment in serum 25(OH)D concentration in four cross-sectional and six longitudinal cohort studies. In addition, both meta-analyses were affected by remarkable heterogeneity due to the inclusion of studies with non-geriatric subjects (up to 50 years old), and especially by the use of different frailty identification tools.

The meta-analyses performed in the present study have a number of strengths, including the use of cross-sectional studies only (n = 13; for the longitudinal studies, only baseline values were included), the selection of more homogeneous studies by age (≥60 years old), and especially the inclusion of studies which identify frailty using only Fried’s criteria. The use of absolute values of 25(OH)D concentration, instead of classes, added specificity to our results. Finally, the two-by-two comparison of non-frail, pre-frail and frail subjects by level of 25(OH)D provided quantitative estimates of effect which supported the hypothesis of a decrease of 25(OH)D being associated with the severity of frailty.

In spite of the limitations discussed above, the results of the two previous systematic reviews and meta-analyses point in the same direction of the current study, indicating the presence of a significant association between lower concentrations of 25(OH)D and frailty. Nevertheless, it is still unclear whether vitamin D deficiency is involved in frailty development or is a consequence of it: frail people present with low muscle strength and have reduced physical activity; hence, they spend less time outdoors, limiting their exposure to sunlight, and their diet is frequently low-quality and unbalanced.

The vitamin D receptor is expressed in the nucleus of muscle cells [58], and vitamin D has been shown to affect muscle cell contractility [59] and increase the de novo synthesis of protein, regulating muscle strength [60]. The number of vitamin D receptors in several organs, including muscle tissues, decreases with age, contributing to reduced muscle strength in later life. Current and previous findings are in accordance with several studies that reported an association between low serum 25(OH)D levels, poor physical performance and low muscle strength [61,62], and loss of muscle mass and sarcopenia [63], which are individual components of the frailty phenotype. In fact, evidence from randomized clinical trials seems to support a beneficial effect of vitamin D supplements in older adults on muscle strength and function (reviewed in [64]) and on physical performance as assessed by timed up and go test [65]. For this purpose, a daily dose of 1000 IU (25 µg/day; 1 µg = 40 IU) appears to be sufficient to obtain significant improvements (enhanced skeletal muscle strength, improvement in physical performance tests, increase in muscle fibers). In contrast, large intermittent doses of vitamin D do not appear to be efficient at improving muscle strength (reviewed in [66]). Falls and fractures are among the main frailty-related health outcomes. In this regard, several meta-analyses [67,68] provided evidence that a supplement of vitamin D combined with calcium (which has a critical structural role, comprising a substantial proportion of the skeleton) results in a 15–20% reduction of hip or non-vertebral fractures in older participants with vitamin D deficiency, meanwhile supplementation with vitamin D alone does not seem to prevent fractures or falls, or have clinically meaningful effects on bone mineral density [69].

As the vitamin D receptor is expressed on immune cells (B cells, T cells, and antigen presenting cells, such as macrophages and dendritic cells), vitamin D plays an important role in the modulation of the innate and adaptive immune response [70,71]. It regulates the production of inflammatory cytokines and immune cells, which are crucial for the pathogenesis of many immune-related diseases [72]. It has been demonstrated that increased concentrations of inflammatory cytokines are associated with frailty status in older adults [73,74], indicating that frail subjects present an additional degree of chronic inflammation with respect to the normal ageing process. Therefore, vitamin D deficiency could act as an intermediate in the relationship between frailty development and an exacerbated inflammatory response. However, evidence from the studies included in a systematic review by Agbalalah et al. [75] did not demonstrate that vitamin D supplementation in adults results in an improvement in circulating inflammatory function biomarkers. Still, this review was not focused on older adults, but mean age of participants in the studies analyzed ranged between 24.8 and 78.8 years. This is an important factor to consider, since only older adults are expected to present a state of low-grade chronic inflammation (the so called “inflammaging”), and hence it is likely that only this population subgroup experiences benefits from vitamin D supplementation.

Vitamin D receptors are also located in the human cortex and hippocampus [76], which are key areas for cognitive function, and their absence has been associated with neurodegenerative dementia such as Alzheimer’s disease [77]. A systematic review by Annweiler et al. [78] concluded that the association between serum 25(OH)D concentrations and cognitive performance is not yet clearly established, and intervention studies investigating the effect of vitamin D supplementation on cognitive functions have obtained inconsistent results [79,80]. The authors suggested that vitamin D insufficiency may negatively affect specific cognitive functions, such as explicit episodic memory, although this conclusion requires further investigations. Physical frailty is often associated with cognitive dysfunction in older adults [81], possibly because of a common underlying pathophysiological and phenotypical basis. The concept of “cognitive frailty” was proposed to emphasize the important role of brain ageing [82]. Cognitive frailty was defined as the simultaneous presence of both physical frailty, operationalized with the Fried’s phenotypical model, and cognitive impairment, diagnosed with a Clinical Dementia Rating scale of 0.5 without a concurrent diagnosis of overt dementia or underlying neurological conditions (see Morley [83] for a review). The possibility that vitamin D deficiency is related to cognitive frailty as well as it is to physical frailty should be investigated in future studies.

There are some limitations in our study. Firstly, seven of the studies collected in the systematic review could not be included in the meta-analyses due to the lack of the necessary comparable data (25(OH)D mean and SD). Nevertheless, since all these studies reported associations between low concentrations of 25(OH)D and frailty status [27,30,34,39,40,41,46], it is likely that present data are not affected by a selection bias. Secondly, sample size was not balanced among the different frailty groups, being frail subjects underrepresented (14%) with regard to pre-frail (42%) and non-frail participants (44%). Thirdly, the number of longitudinal cohort studies was considered too low to perform a meta-analysis including only prospective data to study the relationship between 25(OH)D levels and frailty at follow up, and these studies employed different frailty identification tools. These latter issues, however, may have introduced a non-directional loss of precision and quality rather that generating biased results. Fourthly, heterogeneity among studies was quite high, even in the sensitivity analyses, although it was only a quantitative heterogeneity since almost all studies showed lower level of 25(OH)D in frail and pre-frail subjects. Finally, another limitation may be the language restriction of the literature search; we reviewed only studies written in English or Spanish and may have missed informative studies in other languages. Nevertheless, this issue does not seem to affect met-estimates, given the lack of publication bias demonstrated by Egger’s regression test results.

## 5. Conclusions

In conclusion, the results of the present study indicate the presence of an inverse association between serum 25(OH)D concentration and frailty severity, as defined by the Fried’s phenotype. In view of the current and previous results, considering that vitamin D supplementation is safe and inexpensive, and beneficial effects on the muscular performance have been demonstrated, interventional studies testing the possible benefits of vitamin D supplementation in older adults to prevent/palliate frailty should be considered. A further challenging question is whether vitamin D supplementation in frail subjects may also reduce the negative health outcomes associated with frailty, including dependency and mortality, and whether it may have some effect on cognitive frailty.

## Figures and Tables

**Table 1:** Characteristics of the studies included in the systematic review. Studies in bold were included in the meta-analyses.

Study	First Author Country	Study Design	Population (Mean Age ± SD) (years)	Case Population (Mean Age ± SD) (years)	Control Population (Mean Age ± SD) (years)	Frailty Criteria	Outcome and Assay Method	Results
Alvarez-Ríos et al., 2015 [26]	Spain	Cross-sectional	*n* = 592 female	*n* = 61 frail (median 78, IQR 75–83) *n* = 232 pre-frail (median 74, IQR 71–78)	*n* = 299 non-frail (median 72, IQR 69–76)	Fried’s phenotype	25(OH)D electro-chemiluminescence	↓ 25(OH)D in pre-frail and frail vs. non-frail subjects
Alvarez-Sánchez et al., 2018 [27]	Spain	Cross-sectional	*n* = 631 female (median 74, IQR 70–77)	*n* = 61 frail *n* = 245 pre-frail	*n* = 325 non-frail	Fried’s phenotype	25(OH)D chemiluminescence immunoassay	↓ 25(OH)D with frailty
**Chang et al., 2010** [28]	Taiwan	Cross-sectional	*n* = 215	*n* = 21 frail (72.1 ± 4.4) male/female: 6/15 *n* = 119 pre-frail (71.4 ± 3.8) male/female: 49/70	*n* = 75 non-frail (70.3 ± 3.7) male/female: 32/43	Fried’s phenotype	25(OH)D RIA	↓ 25(OH)D with frailty using Fried’s phenotype
				*n* = 26 frail (71.9 ± 3.7) male/female: 7/19 *n* = 52 pre-frail (71.5 ± 3.6) male/female: 14/38	*n* = 137 non-frail (70.8 ± 4.0) male/female: 66/71	Edmonton Frail Scale		The association between 25(OH)D and frailty is attenuated using the Edmonton Frail Scale
**Ensrud et al., 2010** [16]	USA	Cross-sectional Longitudinal (4.5-year follow-up)	*n* = 6307 female > 69 years old *n* = 4551 female	*n* = 1065 frail (n.p.) *n* = 3047 pre-frail (n.p.)	*n* = 2195 non-frail (n.p.) *n* = 4551 non-frail (non-frail + pre-frail) (n.p.)	Fried’s phenotype	25(OH)D LC-MS/MS	Lower (<20 ng/mL) and higher (≥30 ng/mL) levels of 25(OH)D were moderately associated with higher odds of frailty at baseline Lower levels of 25(OH)D (<20 ng/mL) were modestly associated with an increased risk of incident frailty or death at follow-up
**Ensrud et al., 2012** [29]	USA	Cross-sectional	*n* = 1606 male >65 years old	*n* = 130 frail *n* = 731 pre-frail	*n* = 745 non-frail	Fried’s phenotype	25(OH)D LC-MS/MS	↑ % frail male with ↓ 25(OH)D
**Fried et al., 2009** [30]	USA	Cross-sectional	*n* = 704 female 70–79 years old	*n* = 90 frail *n* = 330 pre-frail	*n* = 284 non-frail	Fried’s phenotype	25(OH)D Radioreceptor assay	↓ 25(OH)D with frailty (nearly significant, *p* = 0.08)
**Gutierrez-Robledo et al., 2015** [31]	Mexico	Cross-sectional	*n* = 331	*n* = 209 frail (78.9 ± 6.4) male/female: 86/123	*n* = 122 non-frail (79.9 ± 4.7) male/female: 66/56	Fried’s phenotype (modified)	25(OH)D ELISA	↓ 25(OH)D in frail vs. non-frail subjects
**Hirani et al., 2013** [32]	Australia	Cross-sectional	*n* = 1511 male 70–79 years old	n.p	n.p	Fried’s phenotype (modified)	25(OH)D 1,25D RIA	↓ 25(OH)D and 1,25D with frailty
Kojima & Tanabe, 2016 [33]	UK	Cross-sectional	*n* = 152 male (70.3 ± 12.8)	*n* = 124 frail (71.9 ± 13.1)	*n* = 28 non-frail (62.8 ± 8.6)	FI (34 items)	25(OH)D n.p	↓25(OH) with frailty
**Krams et al., 2016** [19]	France	Cross-sectional	*n* = 321 (82.94 ± 5.89) male/female: 128/193	*n* = 146 frail (84.10 ± 5.77) male /female: 54/92	*n* = 148 non-frail (81.61 ± 5.59) male/female: 60/88	Fried’s phenotype (modified)	25(OH)D Chemiluminescence immunoassay	25(OH)D levels were not significantly correlated with frailty
Michelon et al., 2006 [34]	USA	Cross-sectional	*n* = 754 female	*n* = 86 frail (mean 75.8, 95% CI 75.1–76.5) *n* = 337 pre-frail (mean 74.4, 95% CI 74.1–74.7)	*n* = 331 non-frail (mean 73.1, 95% CI 73.7–74.4)	Fried’s phenotype	25(OH)D Radioreceptor assay	↓ 25(OH)D with frailty
Navarro-Martínez et al., 2016 [35]	Spain	Cross-sectional	*n* = 104 female (mean = 84)	*n* = 60 Frail *n* = 22 Pre-frail	*n* = 22 non-frail	Fried’s phenotype	25(OH)D GC/MS	↓ 25(OH)D in pre-frail and frail vs. non-frail subjects
Pabst et al., 2015 [36]	Germany	Cross-sectional	*n* = 940 (75.6 ± 6.5) male/female: 478/762	*n* = 38 Frail *n* = 351 Pre-frail	*n* = 551 non-frail	Fried’s phenotype	25(OH)D ECLIA System	↓ 25(OH)D with frailty
Puts et al., 2005 [37]	The Netherlands	Cross-sectional	*n* = 1271	*n* = 242 frail (79.2 ± 6.2) male/female: 91/151	*n* = 1029 non-frail (74.5 ± 6.3) male/female: 531/498	Nine frailty indicators	25(OH)D Competitive binding protein assay	↓ 25(OH)D with frailty
		Longitudinal (3-year follow-up)	*n* = 885	*n* = 125 frail (78.2 ± 6.2) male/female: 56/69	*n* = 760 non-frail (73.4 ± 5.9) male/female: 382/378			↓ 25(OH)D with frailty
**Sanchis et al., 2015** [38]	Spain	Cross-sectional	*n* = 342 male/female: 194/138	*n* = 116 frail (81 ± 7) male/female: 47/69	*n* = 226 non-frail (77 ± 7) male/female: 77/149	Fried’s phenotype	25(OH)D n.p.	↓ vitamin D in frail vs. non-frail subjects 25(OH)D < 9 ng/mL was found as an independent predictor of frailty
Semba et al., 2006 [39]	USA	Longitudinal (3-year follow-up)	*n* = 766 female	*n* = 250 frail (80.4 ± 7.9)	*n* = 516 non-frail (76.1 ± 7.4)	Fried’s phenotype	25(OH)D Radioreceptor assay	↓ 25(OH)D with frailty
**Sergi et al., 2015** [17]	Italy	Cross-sectional	*n* = 1567	*n* = 491 pre-frail (1 positive criterion) (75.18 ± 6.86) male/female: 166/325 *n* = 209 pre-frail (2 positive criteria) (77.77 ± 7.48) male/female: 47/162	*n* = 867 non-frail (71.68 ± 5.55) male/female: 404/463	Fried’s phenotype (modified)	25(OH)D RIA	↓ 25(OH)D with increasing pre-frailty severity
Shardell et al., 2009 [40]	USA	Cross-sectional	*n* = 1005	Male: *n* = 39 frail (80.8 ± 7.8) *n* = 151 pre-frail (76.2 ± 7.1) Female: *n* = 64 frail (82.3 ± 7.4) *n* = 237 pre-frail (76.1 ± 7.5)	Male: *n* = 242 non-frail (71.7 ± 5.5) Female: *n* = 243 non-frail (72.9 ± 6.1)	Fried’s phenotype	25(OH)D RIA	Strong associations of ↓ 25(OH)D with frailty in men and weak association in women
Shardell et al., 2012 [41]	USA	Cross-sectional	*n* = 1005	*n* = 100 frail *n* = 354 pre-frail	*n* = 471 non-frail	Fried’s phenotype	25(OH)D RIA	Participants with ≥20 ng/mL of 25(OH)D had lower prevalence of frailty
**Smit et al., 2012** [18]	USA	Cross-sectional	*n* = 4731	*n* = 453 frail (73.6 ± 0.6) male/female: 140/313 *n* = 1915 pre-frail (72.0 ± 0.4) male/female: 705/1210	*n* = 2363 non-frail (69.4 ± 0.3) male/female: 1099/1264	Fried’s phenotype (modified)	25(OH)D RIA	↓ 25(OH)D with frailty
		Longitudinal (12-year follow-up)						↑ Risk of death with frailty and low serum 25(OH)D
Sousa-Santos et al., 2018 [42]	Portugal	Cross-sectional	*n* = 1447 (mean 74, range 65–100) male/female: 610/873	*n* = 310 frail *n* = 785 pre-frail	*n* = 352 non-frail	Fried’s phenotype	25(OH)D Electro-chemiluminescence immunoassay	↓ 25(OH)D with pre-frailty and frailty
**Tajar et al., 2013** [43]	UK	Cross-sectional	*n* = 1504 male	*n* = 76 frail (72.9 ± 4.7) *n* = 552 pre-frail (70.9 ± 5.6)	*n* = 876 non-frail (68.4 ± 5.5)	Fried’s phenotype (modified) FI (43 items)	25(OH)D RIA	Comparable results using Fried’s phenotype and FI. ↓ 25(OH)D associated with being pre-frail and frail
**Vogt et al., 2015** [44]	Germany	Longitudinal (2.9-year follow-up)	*n* = 727	*n* = 27 frail (n.p.) *n* = 252 pre-frail (n.p.)	*n* = 448 non-frail (n.p.)	Fried’s phenotype (modified)	25(OH)D Chemiluminescence immunoassay	↓ 25(OH)D with incident pre-frailty and combined pre-frailty and frailty
**Wang et al., 2014** [45]	China	Cross-sectional	*n* = 516 male	*n* = 174 frail (81.9 ± 4.4) *n* = 182 pre-frail (74.6 ± 5.2)	*n* = 160 non-frail (72.7 ± 4.1)	Fried’s phenotype	25(OH)D RIA	↓ 25(OH)D levels across frailty categories
Wilhelm-Leen et al., 2010 [46]	USA	Cross-sectional	*n* = 5048 male/female: 2469/2579	n.p.	n.p.	Fried’s phenotype (modified)	25(OH)D RIA	↓ 25(OH)D with frailty
Wong et al., 2013 [47]	Australia	Cross-sectional	*n* = 4203 male (70–88)	n.p.	n.p.	FRAIL scale	25(OH)D Chemiluminescence immunoassay	↓ 25(OH)D with frailty
		Longitudinal (9.2-year follow-up)	*n* = 1625 male		n = 1625 non-frail (FRAIL scale = 0)			↓ 25(OH)D with incident frailty

↑: indicates significant increase (although indicated otherwise); ↓: indicates significant decrease (although indicated otherwise); 1,25D: 1,25-dihydroxyvitamin D; 25(OH)D: 25-hydroxyvitamin D; IQR: interquartile range; CI: confidence interval; ECLIA: enhanced chemiluminescence immunoassay; ELISA: enzyme-linked immunosorbent assay; FI: frailty index; FRAIL: Fatigue, Resistance, Ambulation, Illnesses, & Loss of Weight; GC/MS: Gas chromatography/mass spectrometry; IQR: interquartile range; LC-MS/MS: Liquid chromatography/tandem mass spectroscopy; n.p.: not provided; RIA: radioimmunoassay.

**Table 2:** Summary of meta-analyses comparing frailty groups with assessment of publication bias and sensitivity analysis. Statistically significant *p*-Values are indicated in bold.

Comparison	No. of Studies	Heterogeneity		Meta-Analysis			Publication Bias (Egger’s Test)		Trim-and-Fill		
		I^2^	*p*-Value	SMD	95% CI	*p*-Value	Z	*p*-Value	SMD	95% CI	*p*-Value
All studies											
Frailty vs. non-frailty	12	99.76	<0.0001	−1.31	−2.47, −0.15	**0.0271**	−0.4838	0.6285			
Pre-frailty vs. non-frailty	12	99.83	<0.0001	−0.79	−1.58, −0.003	**0.0491**	−0.3639	0.7159			
Frailty vs. pre-frailty	9	99.61	<0.0001	−0.82	−1.77, 0.13	**0.09**	0.4327	0.6652			
**Sensitivity analysis without Smit et al. (2012)** [18]											
Frailty vs. non-frailty	11	96.05	<0.0001	−0.57	−0.87, −0.28	**0.0002**	−1.3846	0.1662			
Pre-frailty vs. non-frailty	11	88.35	<0.0001	−0.27	−0.38, −0.17	**<0.0001**	−2.5312	**0.0114**	−0.21	−0.31, −0.12	**<0.0001**
Frailty vs. pre-frailty	8	95.55	<0.0001	−0.46	−0.78, −0.14	**0.0048**	−0.4693	0.6388			

CI: confidence interval; SMD: standardized mean difference.

## Supplementary Materials

The following are available online at https://www.mdpi.com/2072-6643/12/8/2286/s1, Table S1. Quality score criteria of the studies included in the meta-analyses.
