# Supplementary material for: Low Vitamin D Levels and Frailty Status in Older Adults: A Systematic Review and Meta-Analysis
Source: Nutrients. 2020 Jul 30;12(8):2286. doi: 10.3390/nu12082286 (PMC7469050; doi:10.3390/nu12082286)
Supplement: Supplementary file 1 [file nutrients-12-02286-s001.pdf]

Table S1. Quality score criteria of the studies included in the meta-analyses. When some data was not available 0 points were assigned

Study reference	Total population size (1)	Age matching (2)	Gender matching (3)	Frailty matching (4)	Frailty measured by specialized personnel (5)	Number of frality groups (6)	Total quality score
Chang et al. (2010) [70]	2	3	2	1	1	2	11
Ensrud et al. (2010) [16]	3	0	1	1	1	2	8
Ensrud et al. (2012) [71]	3	0	1	1	1	2	8
Fried et al. (2009) [63]	3	0	1	1	1	2	8
Gutiérrez-Robledo et al. (2015) [72]	3	3	3	1	1	1	12
Hirani et al. (2013) [73]	3	0	1	1	1	1	7
Krams et al. (2016) [19]	3	3	1	3	1	1	12
Sanchís et al. (2015) [78]	3	3	2	1	1	1	11
Sergi et al. (2015) [17]	3	3	2	1	1	1	11
Smit et al. (2012) [18]	3	3	2	1	1	2	12
Tajar et al. (2013) [80]	3	3	1	1	1	2	11
Vogt et al. (2015) [81]	3	0	3	1	1	1	9
Wang et al. (2014) [82]	3	2	1	3	1	2	12

(1) Less than 100 subjects: 1 point, 100-300 subjects: 2 points, more than 300 subjects: 3 points.
(2) Mean age difference higher than 10 years: 1 point, difference 5-10 years: 2 points, difference lower than 5 years: 3 points.
(3) Rate of one gender either higher than 0.60 or lower than 0.40: 1 point, rate either 0.55-0.60 or 0.40-0.45: 2 points, rate from higher than 0.45 to lower than 0.55: 3 points.
(4) In studies with three frailty groups: 3 points when the rate of none of the groups was lower than 0.3, 2 points when the rate of none of the groups was lower than 0.25, and 1 point in the rest of cases; in studies with two frailty groups: rules the same than those applied to gender-matching.
(5) Absence of description: 1 point, specialization of the personnel not specified or they were not geriatricians or nurses: 2 points, geriatricians or nurses: 3 points.
(6) Only non-frail and frail groups reported: 1 point, a pre-frail group also included: 2 points.
